# Integrating generative AI and reference management tools in assignment writing: insights from an ODeL context

**DOI:** 10.3389/frai.2026.1954585

**Published:** 2026-10-29

**Authors:** Logic Magwa

**Affiliations:** Institute for Open Distance Learning, College of Education, University of South Africa, Pretoria, South Africa

**Keywords:** assignment writing, generative AI tools, integrating, Open and Distance eLearning environment, qualitative case study, reference management tools, Zimbabwe Open University

## Abstract

The rapid advancement of generative Artificial Intelligence (AI) and reference management technologies is transforming academic writing practices in higher education. This qualitative instrumental case study investigates how generative AI and reference management tools are integrated into assignment writing at Zimbabwe Open University within an Open and Distance eLearning (ODeL) environment. Data were collected through semi-structured interviews with seven lecturers and one quality assurance coordinator, as well as focus group discussions with fourteen students selected through purposive sampling. Guided by the Constructivist Learning Theory, the study examined the pedagogical implications of AI-supported writing practices. Findings reveal that tools such as ChatGPT, Grammarly and Mendeley enhance writing quality, research efficiency, accessibility, and learner confidence. However, concerns regarding overreliance, academic integrity and diminished critical engagement were also identified. The study recommends the development of institutional policies, ethical training frameworks and curriculum adjustments to promote responsible and pedagogically sound integration of generative AI tools in ODeL contexts.

## Background

Artificial Intelligence (AI) refers to computer systems capable of performing tasks that typically require human cognitive functions such as learning, reasoning, adaptation and problem-solving (Grajeda et al., 2024). In recent years, the rapid advancement of generative AI technologies has significantly transformed higher education worldwide, enabling tools that can generate human-quality text, code, and data (Mishra and Srivastava, 2024; Marzuki et al., 2023). Tools such as ChatGPT and Grammarly are increasingly adopted by students to support idea generation and language refinement, while reference management tools such as Mendeley and Zotero support citation organisation and management (Segooa et al., 2025).

In Open and Distance eLearning (ODeL) environments, where learners often study independently with limited face to face interaction, generative AI tools can function as digital scaffolds, providing immediate feedback and academic support. This is particularly valuable in resource-constrained contexts where access to physical libraries and real-time academic assistance is limited. Within teacher education programmes, where assignment writing is central to assessment and professional development, AI-supported writing presents both opportunities for enhanced learning and challenges related to overreliance, academic integrity, and diminished critical engagement (Afifah, 2024; West and Allen, 2018).

Despite growing global interest in AI literacy and responsible AI use in academic writing, most studies have been conducted outside Zimbabwe, often in well-resourced higher education contexts (Muthukrishnan et al., 2024; Ateeq et al., 2024). In Zimbabwean institutions including universities like Zimbabwe Open University, the integration of AI in assignment writing is emerging but remains largely unregulated and poorly understood (Segooa et al., 2025). There is a limited empirical understanding of how students and lecturers use these tools and how institutional policies, and pedagogical strategies could support responsible and effective AI integration.

Teacher education warrants specific investigation because it not only develops students’ academic writing competence but also shapes their future pedagogical practices. Teacher education is particularly important because prospective teachers are expected to develop academic, digital, and professional competencies that can subsequently inform their classroom practices (Muthukrishnan et al., 2024). Unlike other disciplines, student teachers are expected to transfer learned academic skills into classroom instruction. Consequently, their use of generative AI tools has implications beyond individual learning, influencing how future educators may integrate or regulate AI in their own teaching contexts. This dual role makes teacher education a critical site for examining the pedagogical and ethical integration of AI.

Given this context, exploring the integration of AI tools in Zimbabwean ODeL settings is timely and necessary. It provides insights into how students navigate assignment writing with AI, the pedagogical implications for teacher education, and the ethical considerations that institutions must address to ensure that AI adoption enhances learning rather than undermines critical thinking or academic integrity (Afifah, 2024; West and Allen, 2018). This study therefore contributes context-specific knowledge from the Global South, informing policy development, curriculum design and ethical frameworks for AI-supported learning in higher education. Guided by this focus, the study was framed by the following research question:

- How are generative AI and reference management tools integrated into assignment writing in an Open and Distance eLearning environment?

## Literature review

### International literature

Recent studies highlight the growing integration of Artificial Intelligence (AI) tools in academic writing across higher education contexts. Afifah (2024) found that AI supports idea generation, structured organisation, and language refinement among graduate students, while Gururaj and Dsouza (2024) reported that AI writing assistants enhance grammar accuracy, coherence, and workflow efficiency, though concerns about overreliance remain. Muthukrishnan et al. (2024), focusing on perspective teachers in Malaysia, emphasised the importance of AI literacy and responsible use in assignment writing. Similarly, Ateeq et al. (2024) demonstrated that AI tools can improve topic selection, content organisation and adherence to academic conventions in university assignments and thesis work.

Floris and Renandya (2025)examined AI integration in research writing and provided practical strategies for improving scholarly output, but their focus was limited to research rather than routine assignment writing. Nguyen (2023) highlights that AI reshapes learning by improving access to information while raising dependency concerns. Song and Song (2023) show that AI improves motivation and writing fluency through feedback mechanisms. Marzuki et al. (2023) confirms improvements in grammatical accuracy through AI writing tools. Khalifa and Albadawy (2024) emphasise productivity gains in citation management and writing efficiency. Bahamman et al. (2023) caution about ethical risks and regulatory challenges in AI-assisted writing. Bhutoria (2022) argues that AI enhances personalised learning but may encourage passive if not properly structured. Gupta and Kaul (2024); emphasise AI as a scaffold within the Zone of Proximal Development when guided by pedagogy. Kadaruddin (2023) highlights that AI supports creativity and critical engagement when used as a structured learning tool.

Despite these contributions, international literature is largely concentrated in well-resourced and conventional higher education settings, with limited attention to Open and Distance eLearning (ODeL) environments where learner autonomy, limited lecturer interaction and digital dependency significantly shape writing practices. This study addresses this gap by providing empirical evidence from Zimbabwean ODeL context, thereby extending constructivist interpretations of AI as both a cognitive scaffold and a potential substitute for independent learning in assignment writing.

### Zimbabwean literature

Within Zimbabwe, Gawe and Gudyanga (2025) explored the impact of AI on development studies students, identifying enhanced engagement alongside concerns about critical thinking. Mukwerete and Matiza (2025) investigated AI integration among tertiary students and highlighted issues of responsibility and accountability in academic writing. However, these Zimbabwean studies address general academic writing practices rather than assignment-specific processes in teacher education or ODeL contexts. This study addresses this gap by investigating how students, lecturers and quality assurance personnel at Zimbabwe Open University integrate AI tools into assignment writing, thereby generating context-specific insights that inform pedagogical practice, assessment design and institutional AI policy development.

From a constructivist perspective, learning is an active process of meaning-making, where tools and interactions support knowledge construction (Mcleod, 2025). Generative AI and reference management tools can therefore be conceptualised as mediational tools that facilitate scaffolding within learners’ Zone of Proximal Development, while also posing risks of cognitive offloading if used uncritically (Gupta and Kaul, 2024; Muthukrishnan et al., 2024).

While existing studies consistently demonstrate the benefits of generative AI and reference management tools in enhancing writing quality and research efficiency, there is limited synthesis regarding how these tools influence cognitive engagement and knowledge construction. Moreover, prior research tends to emphasise technological affordances without sufficiently interrogating pedagogical implications within specific disciplinary and contextual settings. This study addresses this gap by situating AI integration within a constructivist framework and focusing specifically on assignment writing in teacher education within an ODeL context.

## Statement of the problem

Although generative Artificial Intelligence (AI) tools such as ChatGPT, Grammarly and reference management tools (Mendeley, Zotero) are increasingly being used by students to support academic writing, there is limited scholarly evidence on how these tools are pedagogically integrated into assignment writing within Open and Distance eLearning (ODeL) institutions in Zimbabwe. Existing studies on AI in education have largely been conducted outside Zimbabwe and predominantly in conventional university settings, focusing on general academic writing, AI literacy or research-related practices (Ateeq et al., 2024; Muthukrishnan et al., 2024; Nguyen, 2023; Song and Song, 2023). Consequently, little is known about how students and lecturers in Zimbabwean ODeL environments utilise generative AI tools and reference management tools in assignment writing, the benefits they associate with these tools, the challenges they encounter and how such use aligns with pedagogical principles such as Constructivist Learning Theory. This creates a contextual evidence gap because findings from other educational settings may not fully reflect the technological and institutional conditions of Zimbabwean ODeL environments. Context-specific evidence can therefore support the development of institutionally relevant policies, ethical guidelines, and curriculum strategies for responsible AI integration. Therefore, the purpose of this study is to explore how generative AI tools and reference management tools are integrated into assignment writing at Zimbabwe Open University to examine the perceived benefits and challenges from the perspectives of students and lecturers and to generate insights that can inform pedagogically sound and ethically responsible AI integration in ODeL environments.

## Theoretical framework

This study draws from Constructivist Learning Theory, which emphasises that knowledge is actively constructed by learners through interaction with their environment rather than passively received from external source (Mcleod, 2025). This theory emphasises learner-centred, inquiry driven and contextually rich environments that promote critical thinking, autonomy, and knowledge construction. This perspective is supported by existing literature which emphasises that learning occurs through guided interaction and scaffolding within socially mediated contexts (Vygotsky, 1978; Brunner, 1985). Furthermore, recent studies highlight those digital tools, including AI can function as cognitive scaffolds when aligned with constructivist principles (Muthukrishnan et al., 2024; Ateeq et al., 2024). In assignment writing, generative AI tools such as ChatGPT and Grammarly can serve as scaffolding tools that support learners throughout the writing process by helping them generate ideas, organise their thoughts and improve grammatical accuracy (Muthukrishnan et al., 2024; Ateeq et al., 2024). Reference management tools such as Mendeley support citation organisation and should be distinguished from Generative AI tools. These tools help students to generate ideas, organise their thoughts, correct grammatical errors and manage citations. This aligns with the constructivist view that learning is enhanced when students are supported in engaging with tasks that are slightly beyond their current capabilities that is Zone of Proximal Development (ZPD) (Gupta and Kaul, 2024). AI-driven tools can dynamically adjust to the learner`s zone of proximal development, enhancing educational outcomes for students with varying abilities. Within this framework generative AI and reference management tools can be conceptualised as cognitive scaffolds that support learners within ZPD. However, if learners depend excessively on AI-generative outputs without reflective engagement, the scaffolding function may shift towards cognitive substitution, whereby contradicting constructivist principles. This study therefore interprets findings through the dual lens of scaffolding and learner autonomy.

## Conceptual framework

The conceptual framework presented in Figure 1 illustrates how Constructivist Learning Theory underpins the integration of generative AI and reference management tools in assignment writing within an Open and Distance eLearning (ODeL) environment. Guided by the principle of scaffolding and the Zone of Proximal Development (ZPD), AI tools such as ChatGPT, Grammarly, Mendeley, Zotero and ProWriting Aid provide academic support by enhancing writing quality, facilitating research, and improving accessibility and efficiency. These functions contribute to improved assignment-writing outcomes, including better organisation, clarity, confidence, and productivity. However, the framework also recognises that overreliance on AI may lead to academic integrity concerns, reduced critical thinking, cognitive offloading, and diminished learner autonomy. Consequently, the framework emphasises that responsible and ethical AI integration, supported by institutional policies, AI literacy programmes, reflective learning practices and assessment redesign, is necessary to ensure that AI serves as a cognitive scaffold rather than a substitute for independent knowledge construction.

**Figure 1:**
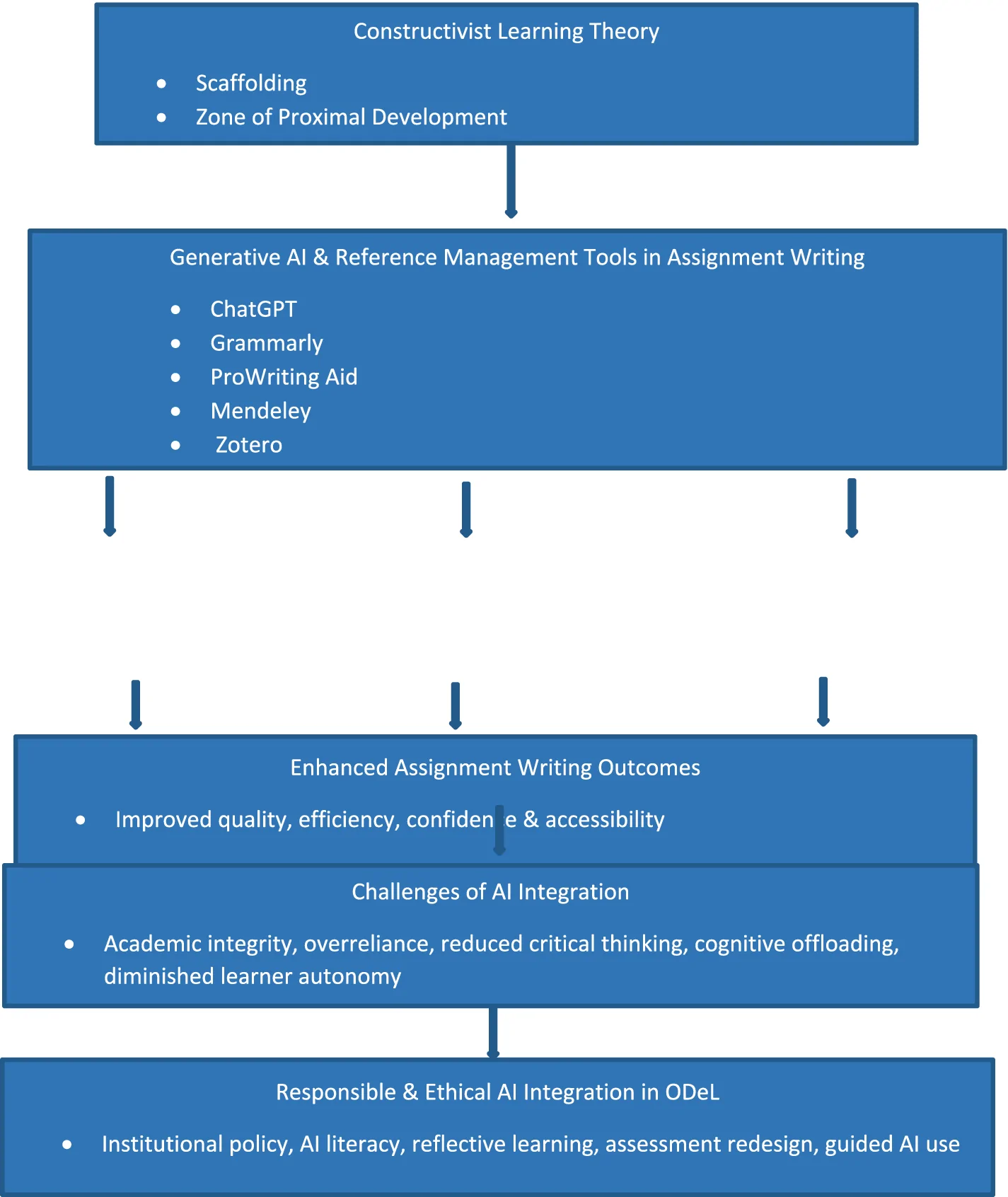
Conceptual framework for the integration of generative AI and reference management tools in assignment writing within ODeL environment.

## Methods

This study adopted a qualitative instrumental case study design to explore how generative artificial intelligence and reference management tools are being used to support assignment writing within an Open and Distance eLearning context (Magwa and Magwa, 2015). The Masvingo Regional Campus of Zimbabwe Open University was selected as an instrumental case because it represents a typical regional ODeL campus with diverse student demographics, active use of digital learning platforms and exposure to both institutionally supported and learner-initiated AI tools. The case was intended to illuminate broader issues surrounding AI-supported assignment writing in resource-constrained ODeL environments rather than to generate statistically generalisable findings.

To enhance methodological transparency, detailed procedures were followed throughout the research process. Participants were selected through purposive sampling based on their experience with AI-supported learning. Data collection was conducted using semi-structured interviews that lasted between 45 and 60 min with seven lecturers and one quality assurance coordinator and focus group discussions for a duration of 70–90 min with fourteen students (see Table 1).

**Table 1:** Participants’ details.

Category	Number (*n*)	Inclusion criteria	Data collection participation
Students	14	Two students representative from each faculty male and female	Focus group
Lecturers	7	Male and female with more than five years of teaching experience. Two from each faculty	Semi-structured interviews
Quality assurance coordinator	1	They are deemed to have knowledge and experience gained by working with students and lecturers	Semi-structured interviews

The generated data was transcribed into text, and a thematic content analysis, relying on identifying and analysing the emerging themes from all the forms of data that were collected, was used. Following this logic, the researcher was able to link the various opinions of participants on how AI can be effectively used to support assignment writing in ODeL environments.

The study's potential for rich and important data determined participant selection. The sample size was considered adequate for this qualitative case study because participants were purposively selected for their direct experience with AI-supported learning and assignment writing. Adequacy was therefore guided by information richness and the extent to which the interviews and focus group discussions generated sufficient depth and recurring patterns to address the research questions. Table 1 lists the participants' details.

## Ethical considerations

Ethics approval was obtained from the University of South Africa’s College of Education, Research Ethics Committee (Reference no 7673) after meeting privacy, confidentiality, informed consent, assent, and voluntary participation requirements. Before conducting the study, permission was sought from the Ministry of Higher and Tertiary Education, Innovation, Science and Technology Development to undertake fieldwork at Zimbabwe Open University, Masvingo Regional Campus. The purpose of the study was explained to the participants. The participants were informed that they could withdraw from the study at any time without consequences. Friedriksen and Rhodes (2004) aver that informing the participants of their right to withdraw from a study is an important aspect of the research process. Member checking processes were conducted after the analysis process and contributed to the rigour and trustworthiness of the findings. The quality assurance criteria of dependability, conformability, transferability, and credibility were employed.

## Data analysis

The researcher used Braun and Clarke’s (2006, p. 87) six phases of thematic analysis. Interview transcripts were read and re-read to align with the first step or familiarization of the data. In the second step, the data were coded. In this case, coding schemes were used to simplify the correlations between the data. In the third instance, patterns and repetitions in the data sets were recorded. In the fourth step the emerging themes were reviewed to check if they work in relation to the coded extracts. Themes were then refined to generate clear names in the fifth step and finally, the recurring patterns were collated into themes. The researcher coded the data independently and developed the themes. Themes were reviewed and refined to ensure coherence and alignment with the research question and theoretical framework. To enhance trustworthiness, emerging themes were compared across data sources and reflective memo-writing was used to minimize researcher bias.

Initial codes were generated, grouped into categories, and refined into three overarching themes. The prevalence of themes was determined based on frequency of occurrence across interviews and focus group discussions, as well as the depth and emphasis placed on them by participants.

## Results

The presentation of results is guided by Constructivist Learning Theory, particularly the concepts of scaffolding and the Zone of Proximal Development which frame the interpretation of how generative AI and reference management tools support or potentially constrain student learning. Results are presented according to three themes:

- Academic Support and Writing Enhancement
- Research and Content Development
- Practical Efficiency and Accessibility

### Academic support and writing enhancement

The study's participants stressed that in the artificial intelligence era generative Artificial Intelligence (AI) enhances academic support and writing. This theme encompasses improved writing quality such as grammar, clarity and structure, real-time personalised learning and confidence building in academic work, all of which lead to improved academic support and writing enhancement.

In support of the above, one of the lectures during interviews had this to say:

*AI tools are a rich knowledge reserve. Students access the desired information with easy. There is no wastage of time (L1)*.

The above quote implies that generative AI tools make it easy for students to get more information and knowledge quickly and efficiently. Another lecturer during an interview session confirms the effective of AI as follows:

*AI tools are models for standard academic English, however, some lecturers cautioned that when students rely heavily on AI-generated language and disciplinary jargon, they may reproduce polished text without fully understanding the underlying concepts, potentially weakening critical engagement and authentic voice development in assignment writing (L5)*.

While AI supports exposure to academic language, overreliance may hinder students’ deep understanding and development of a personal academic voice, suggesting a need for guided use.

Similarly, one of the students narrated her experience as follows:

*I benefited greatly from AI tools during my teaching practice. Although our teaching practice school had no library, I was able to complete my assignments effectively with the help of AI resources (S8)*.

A quality assurance coordinator added that:

*Through AI tools, students have the advantage of refining generated content, which provides a useful starting point. However, beginning with AI-produced drafts may reduce the cognitive struggle associated with constructing ideas independently, potentially shifting AI from a scaffold within the Zone of Proximal Development to a substitute for active knowledge construction (QA1)*.

AI can scaffold writing by providing a starting point, but excessive reliance risks replacing independent thinking, highlighting the importance of balancing support with active engagement.

Across participant groups, there was broad agreement that generative AI tools enhance academic writing by improving grammar, language clarity, organisation, and learner confidence. Students primarily emphasised the immediate academic support and accessibility offered by AI tools, particularly in resource-constrained contexts such as teaching practice, whereas lecturers acknowledged these benefits while expressing concerns about students’ overreliance on AI-generated language and disciplinary terminology. The quality assurance coordinator adopted a more balanced institutional perspectives by recognising AI as a useful starting point for writing while cautioning against the replacement of independent cognitive effort. These contrasting perspectives reveal a clear tension between efficiency and authentic learning. From a constructivist perspective, the findings suggest that generative AI functions as a cognitive scaffold within learners’ Zone of Proximal Development by supporting tasks that students may initially struggle to complete independently. However, when learners depend excessively on AI -generated outputs without critically interrogating or adapting them, the scaffolding process shifts towards cognitive substitution, thereby limiting opportunities for independent knowledge construction and the development of an authentic academic voice.

### Research and content development

According to the results of the study generative Artificial Intelligence (AI) tools facilitate greatly in research and content development. AI tools enhance content quality through expressing complex ideas more clearly and effectively. They also assist in brainstorming ideas, offering quick ideas and suggestions.

In line with the above facts a lecturer during interviews expressed her sentiments as follows:

*Generative AI tools are perceived as resource reserves that provide quick access to related literature; however, our concern is that students may rely on AI-generated summaries rather than engaging directly with primary scholarly sources, thereby limiting exposure to nuanced arguments and methodological diversity (L2)*.

AI improves efficiency in accessing literature but may reduce critical interaction with primary sources, emphasising the need for students to actively engage with original research.

Another lecturer added that:

*Through generative AI tools data is readily available and can be accessed in whatever form, pictorial, maps, population, charts or even statistics (L6)*.

This suggests that AI tools provide access to a relevant information for research to a particular topic. A student confirms the importance of generative AI tools in research and content development as follows:

*For us students AI tools assist in finding research articles that are in line or closely related and relevant to an area of study. Giving background information on the topics (S4)*.

A quality assurance coordinator confirms that:

*AI tools help students save time by summarising articles and gathering educational theories, nevertheless, excessive dependence on AI-assisted summarisation may compress complex academic debates into simplified outputs, reducing opportunities for critical analysis and independent synthesis of scholarly perspectives (QA1)*.

Time-saving benefits are evident, yet overuse of AI summaries can simplify complex ideas, potentially undermining students’ analytical and synthesising skills.

Participants consistently acknowledged the contribution of generative AI to research efficiency, although they differed in the aspects, they considered most significant. Students valued AI primarily for facilitating access to relevant literature and generating background information, while lecturers were more concerned about the tendency of students to rely on AI-generated summaries rather than consulting original scholarly sources. The quality assurance coordinator similarly recognised the time saving benefits of AI but questioned whether summarised outputs might reduce students’ capacity for critical synthesis and evaluation of academic literature. These findings demonstrate an important tension between improved research efficiency and the maintenance of scholarly rigour. Within Constructivist Learning Theory, meaningful learning occurs when students actively engage with diverse perspectives and construct their own understanding. Although AI tools can scaffold literature exploration and information retrieval, excessive dependence on AI-generated summaries may restrict learners’ opportunities to analyse competing viewpoints, evaluate evidence critically and construct knowledge independently.

### Practical efficiency and accessibility

It was established that AI helps student teachers to meet their deadlines in assignment writing using tools like Zotero for formatting, ChatGPT for drafting and ProWritingAid for editing. This theme encompasses time management, anytime access to academic support, personalised learning, and independent improvement.

In support of the above one of the lecturers indicated that:

*AI tools offer automatic editing and formatting, allowing student teachers to concentrate on content and save time, however, my concern is that this efficiency may obscure the depth of students’ cognitive engagement, making it difficult to determine whether improvements reflect genuine skill development or technological assistance (L4)*.

Efficiency gains are clear, but automatic corrections may mask whether students are truly developing editing and writing skills independently. In support of these AI tools in assignment writing one of the lecturers in an interview session added that:

*These tools promote accessibility through the provision of simple language explanations for better understanding (L3)*.

This indicates that AI tools provide clear and understandable information that make it easier for students to access relevant data. Another lecturer during interviews confirms the need for AI tools on assignment writing:

*Incorporating generative AI in assignment writing this helps to improve individual learning. This individual learning helps students to grow in academic processes independently (L7)*.

One of the students during the focus group discussion indicated that:

*We access information easily on mobile phones wherever there is network and covers all areas (S2)*.

Another student added that:

*Through using our mobile phones AI tools provides full access to a comprehensive collection of educational resources or materials that may otherwise be difficult to obtain (S14)*.

Similarly, a quality assurance coordinator had this to say:

*Generative AI assists in the rapid generation of ideas, enabling students to refine content more efficiently, however, my worry is when AI consistently provides structured drafts and instructional guidance, it may reduce opportunities for iterative drafting, reflective questioning, and independent meaning-making, raising concerns about long-term academic autonomy (QA1)*.

AI accelerates idea generation and personalisation, yet overreliance may limit reflective practice and long-term autonomy in learning and writing.

Participants shared the view that generative AI enhances efficiency by enabling students to complete assignments more quickly and access academic support regardless of time or location. Students particularly valued the convenience of mobile access and the availability of educational resources, whereas lecturers acknowledged these practical advantages but questioned whether automated editing and drafting tools adequately promote long-term writing competence. The quality assurance coordinator further highlighted concerns that continual reliance on AI-generated drafts could reduce opportunities for reflection, iterative writing and independent problem solving. These differing perspectives illustrate the balance that institutions must achieve between technological efficiency and sustainable learning. Viewed through a constructivist lens, AI tools provide valuable scaffolding that supports learners in completing complex academic tasks. However, constructivism also emphasises the gradual withdrawal of support as learners develop competence and autonomy. The findings therefore suggest that while AI can enhance accessibility and productivity, its educational value depends on structured pedagogical guidance that encourages students to engage critically with AI-generated outputs rather than simply accepting them.

Despite the reported benefits, participants expressed concerns that excessive reliance on AI-generated drafts may undermine originality, critical thinking, and independent writing development. Some lecturers indicated difficulties in distinguishing between authentic student work and AI-assisted submissions.

The above findings align with constructivist principles, where AI tools function as scaffolds supporting learners within their Zone of Proximal Development, while also raising concerns about reduced independent knowledge construction when overused.

## Discussion

The discussion is grounded in empirical findings of the study, with interpretations explicitly linked to participant responses and thematic patterns identified during analysis. Furthermore, the discussion interprets the findings in relation to Constructivist Learning Theory and existing scholarship on AI -supported academic writing, highlighting both pedagogical affordances and emerging tensions within ODeL environments. The findings demonstrate that generative AI tools function primarily as cognitive scaffolds that enhance writing quality and research efficiency. Consistent with Constructivist Learning Theory, these tools appear to support learners within Zone of Proximal Development by providing immediate feedback, idea generation and structural modelling. As illustrated in Figure 1, these affordances are positioned within a broader constructivist framework in which AI and reference management tools can support academic development while requiring guided use to preserve learner autonomy and independent knowledge construction. However, the findings also reveal an emerging tension between technological efficiency and academic integrity, particularly where excessive reliance on AI-generated outputs may reduce critical engagement and independent knowledge construction.

The findings align with prior research indicating that AI tools can enhance writing organisation, coherence, vocabulary, and grammatical accuracy (Ateeq et al., 2024; Khalifa and Albadawy, 2024; Song and Song, 2023). Studies further indicate that AI powered writing tools including Grammarly and ChatGPT can enhance linguistic precision and structural clarity (Khalifa and Albadawy, 2024). In addition, generative AI tools can support brainstorming, content structuring and critical engagement with academic tasks (Kadaruddin, 2023; Muthukrishnan et al., 2024). Collectively, this body of literature confirms that AI integration has the potential to improve efficiency, creativity, and accessibility in assignment writing. Beyond improving surface-level linguistic accuracy, the findings suggest that generative AI tools may be reshaping epistemic engagement in assignment writing. While students benefit from grammar correction and structural modelling, there is risk that algorithmically generated coherence may mask shallow conceptual understanding. For example, participants’ emphasis on improved writing quality and efficiency directly reflects the role of AI as a cognitive scaffold, while concerns about overreliance illustrate the potential shift toward cognitive substitution, as evidenced in the results section. In a constructivist framework, scaffolding is intended to gradually withdraw as learner competence increases. However, if AI tools continuously supply refined language and structure without requiring cognitive struggle, the scaffolding may become permanent rather than transitional. This shifts AI from a supportive mediator within the Zone of Proximal Development, ZPD to a substitute for independent meaning-making. Furthermore, in the ODeL context, where lecturer-student interaction is limited, AI tools may inadvertently replace dialogic feedback processes that are central to constructivist pedagogy. Instead of negotiated meaning between lecturer and student, feedback becomes automated and transactional. While this increases efficiency, it may reduce opportunities for critical dialogue, reflective questioning, and deeper conceptual clarification. This tension raises important pedagogical questions. Are students internalising academic conventions through AI-supported modelling or are they externalising cognitive effort to technological systems? The findings suggest that while AI enhances writing presentation, institutions must design structured reflective activities that ensure students actively interrogate AI-generated suggestions rather than passively adopting them.

Consistent with Zimbabwean research Mukwerete and Matiza (2025) reported that AI tools enhance research efficiency and assist students in refining topics, identifying grammatical errors, and improving overall academic presentation. Similarly, Khalifa and Albadawy (2024) emphasised the value of citation management systems such as Zotero and Mendeley in organising research materials. While these tools streamline research processes, the present findings caution that efficiency must be accompanied by strengthened information literacy to prevent superficial engagement with scholarly sources. While participants celebrated the efficiency gains in literature identification and summarisation, the findings reveal a potential epistemological shift in how students engage with research. AI-assisted summarisation may compress complex scholarly debates into simplified outputs, potentially reducing students’ exposure to nuanced argumentation and methodological diversity. In constructivist terms, authentic knowledge construction requires learners to grapple with competing perspectives and interpret primary sources. When AI tools pre-process information, there is a risk that students engage with synthesised interpretations rather than original scholarship. Moreover, the perception of AI as a resource reserve may foster an illusion of comprehensiveness. Generative AI systems do not access live databases in real time and many produce incomplete, biased or fabricated references. In resource-constrained ODeL environments, where access to subscription databases is already limited, overreliance on AI-generated literature may further narrow scholarly engagement instead of expanding it. The findings therefore highlight a paradox: AI tools increase research accessibility while simultaneously necessitating stronger information literacy competencies. Without training in source verification, citation validation and critical evaluation of AI outputs, research efficiency may come at the expense of academic rigour.

The efficiency gains identified in this study are particularly significant within ODeL environments characterised by time constraints and geographical dispersion. Prior research similarly indicates that AI tools facilitate idea generation, editing and proofreading (Mukwerete and Matiza, 2025; Bahamman et al., 2023), increase writing speed (Gururaj and Dsouza, 2024) and personalise learning experiences (Ateeq et al., 2024; Bhutoria, 2022). However, while AI-supported accessibility through mobile devices democratises academic support, variations in digital literacy and internet connectivity may produce uneven benefits among students. Thus, technological inclusion does not automatically guarantee pedagogical equity. AI tools appear to function as compensatory academic support mechanisms in contexts where physical libraries, writing centres and immediate lecturer feedback are limited. However, efficiency should not be conflated with educational depth. Rapid drafting and automated formatting may compress the recursive nature of the writing process, reducing opportunities for iterative and metacognitive monitoring. This concern is consistent with process-based perspectives of writing, which emphasise planning, drafting, monitoring and revision as interconnected stages of knowledge construction. Additionally, accessibility through mobile devices democratises academic support, yet it simultaneously exposes structural inequalities. Variations in internet connectivity, device quality and digital literacy may produce uneven benefits among students. These inequalities are also documented in ODeL literature, where differences in connectivity, access to suitable devices and digital literacy can shape students’ participation in technology-mediated learning. Thus, while AI tools promote inclusion in principle, that pedagogical impact may differ across socio-economic contexts within Zimbabwe’s ODeL landscape. The difficulty lecturers reported in distinguishing authentic from AI-assisted work also signals a broader assessment dilemma. Traditional take-home assignments may no longer accurately measure individual cognitive performances in AI-pervasive environments. This suggests a need to redesign assessment practices toward process-oriented evaluation, oral defenses, reflective commentaries and staged submissions that foreground learner reasoning rather than final textual polish. This recommendation is consistent with emerging literature advocating process-oriented and authentic assessment approaches that emphasise learner reasoning, reflection, and evidence of the learning process rather than reliance on the final product.

Taken together, the findings reveal that generative AI tools function simultaneously as cognitive scaffolds, efficiency enhancers and potential disruptors of epistemic agency. Within Constructivist Learning Theory, effective scaffolding supports learners toward autonomy. However, autonomy requires gradual internalisation of skills rather than sustained technological dependency. The pedagogical challenge for ODeL institutions is therefore not whether to integrate generative AI tools, but how to structure their use in ways that preserve cognitive engagement, ethical integrity, and critical inquiry. Responsible integration demands institutional policy frameworks, AI literacy development and assessment redesign that align technological affordances with constructivist principles of active knowledge construction.

## Conclusion

This study contributes to emerging scholarship on AI integration in teacher education by providing empirical evidence from an ODeL university context, an area that remains under researched. While findings highlight the pedagogical benefits of generative AI tools, the study also underscores the need for structured institutional frameworks to mitigate risks.

### Recommendations

The study recommends the development of comprehensive institutional AI policies that clearly define acceptable and unacceptable uses of generative AI in academic work. Additionally, structured AI literacy programmes should be introduced to equip both students and lecturers with skills for critical evaluation and responsible use of AI tools. Curriculum design should be adjusted to incorporate reflective and process-based assessment methods.

### Limitations

This study has several limitations. First, the findings are based on self-reported data, which may be subject to bias or inaccuracies in participant recall. Second the study was conducted at a single institutional site, limiting the generalizability of the findings to other ODeL contexts. Third, the use of a single coder during data analysis may introduce subjectivity in theme identification, although reflective memo-writing was employed to enhance consistency. These limitations suggest that findings should be interpreted with caution. Future research should consider multi-site studies, the use of multiple coders and mixed methods approaches to enhance validity, reliability and generalizability.

## Data Availability

The original contributions presented in the study are included in the article/supplementary material, further inquiries can be directed to the corresponding author.
